# Supplementary material for: WordCommentsAnalyzer: A windows software tool for qualitative research
Source: F1000Res. 2018 Sep 4;7:536. Originally published 2018 May 3. [Version 2] doi: 10.12688/f1000research.14819.2 (PMC6137411; doi:10.12688/f1000research.14819.2)
Supplement: Supplementary file 3 [file f1000research-7-17614-s0002.tgz › b2a79496-4ada-47c4-9682-a99ce867836d.zip!/Eight Iranian Health Journals/The Journal of Toloo-e-Behdasht_11.docx]

© The Journal of Toloo-e-Behdasht. This work is licensed under CC BY 4.0 (https://creativecommons.org/licenses/by/4.0/).

Volume & Issue: دوره 15 - شماره 3

Title: آگاهی رابطان بهداشتی شهرستان دماوند در خصوص ایمنی غذایی: یک مطالعه مبتنی بر راهنمای سازمان جهانی بهداشت

http://tbj.ssu.ac.ir/article-1-2266-fa.html

مقدمه: بیماری‌های منتقله از غذا معمولاً به دلیل آلودگی میکروبی یا سموم موجود در طبیعت ایجادشده و با ورود این میکروارگانیسم‌ها (باکتری‌ها، ویروس‌ها، انگل‌ها) و یا مواد شیمیایی از طریق آب و غذای آلوده به انسان منتقل می‌شود. مواد غذایی ناسالم زمینه‌ساز بیش از 200 بیماری از بیماری‌های اسهالی تا انواع مختلفی از سرطان‌ها می‌باشند. لذا ایمنی مواد غذایی یک اولویت بهداشت عمومی است. مطالعه حاضر باهدف تعیین میزان آگاهی رابطان بهداشتی شهرستان دماوند درباره ایمنی غذایی با توجه به راهنمای سازمان جهانی بهداشت انجام‌گرفته است. روش بررسی: مطالعه مقطعی حاضر به‌صورت توصیفی- تحلیلی می‌باشد که گروه هدف آن، رابطان بهداشت مراکز بهداشتی درمانی شهرستان دماوند از توابع دانشگاه علوم پزشکی شهید بهشتی بوده است. تعداد کل شرکت‌کنندگان در این بررسی 109 نفر بود که به روش سرشماری و با رضایت داوطلبانه و آگاهانه وارد مطالعه گردیدند. ابزار جمع‌آوری داده‌ها، پرسشنامه محقق ساخته شامل 13 سؤال در مورد مشخصات دموگرافیک و 24 سؤال در خصوص ایمنی غذایی بر مبنای راهنمای سازمان جهانی بهداشت بود. داده‌ها به کمک نرم‌افزار SPSS-18 و آزمون‌های آماری آنالیز واریانس یک‌طرفه، تی تست و همبستگی اسپیرمن تجزیه‌وتحلیل گردید. یافته­ها: تعداد رابطان شرکت‌کننده 109 نفر با میانگین سنی 98/9±75/44 سال بود. میانگین نمره آگاهی رابطان در خصوص ایمنی غذایی (5 کلید دستیابی به غذای سالم‌تر و ایمن‌تر) 22/6±87/35 بود. سطح آگاهی 77 نفر (71 درصد) از رابطان در حد خوب بود. در این مطالعه بین آگاهی با وضعیت تأهل، سن، تحصیلات، سابقه کار، شغل و تحصیلات همسر رابطه معنی‌داری دیده نشد (05/0<P) بحث و نتیجه‌گیری: آگاهی رابطان در مورد برخی آیتم‌های ایمنی غذایی در حد خوب بود و در برخی موارد مانند نحوه نگهداری مواد غذایی، راه‌های انتقال میکروب، درجه حرارت مناسب برای پخت گوشت و غیره آگاهی کمی وجود دارد که در این رابطه اجرای برنامه‌های آموزشی جهت بالا بردن اطلاعات و آگاهی‌های جایگاه رابطان بهداشتی پیشنهاد می‌شود.

Title: مقایسه وضعیت روی در بیماران مبتلابه فنیل کتونوری تحت درمان با فرمولای متابولیک با افراد سالم

http://tbj.ssu.ac.ir/article-1-2268-fa.html

مقدمه: غذاهای غنی از پروتئین در بیماران مبتلابه فنیل کتونوری محدود می‌شوند. فرمولای متابولیک بدون فنیل الانین جایگزین شیر، فرآورده‌های لبنی، گوشت و ماهی می‌شود. لذا این بیماران ممکن است در معرض خطر کمبود پروتئین و درنتیجه کمبود روی قرار گیرند. هدف از این بررسی تعیین وضعیت روی در بیماران فنیل کتونوری تحت درمان با فرمولای متابولیک در مقایسه با افراد سالم بود. روش و بررسی: این مطالعه مورد – شاهدی با مشارکت 29 بیمار مبتلابه فنیل کتونوری (PKU) و 29 نفر فرد سالم به‌عنوان شاهد با سن 42-5/0 سال در استان یزد و بوشهر انجام شد. غذاهای دریافتی روزانه به‌وسیله پرسشنامه یادداشت خوراک به مدت 3 روز گردآوری گردید.  غلظت روی پلاسما با روش اسپکتروفتومتری با جذب اتمی شعله‌ای اندازه‌گیری شد.  p کمتر از 05/0 به‌عنوان سطح معنی‌داری در نظر گرفته شد. یافته‌ها: میانه‌روی پلاسما در گروه مورد و شاهد به ترتیب 86/5 و 34/8 میکرومول بر لیتر بود (11/p<.). میانه‌روی پلاسما در هر دو گروه کمتر از دامنه نرمال بود. روی دریافتی در گروه مورد نسبت به کنترل به‌طور معنی‌داری بیشتر بود (001/p<.).فراوانی کمبود روی در گروه مورد 82.8 و در گروه شاهد 86.2 درصد و در دو گروه تفاوت معنی‌داری نداشت (1p=). نتیجه‌گیری: باوجوداینکه دریافت روی در گروه مورد نسبت به شاهد به‌طور معنی‌داری بیشتر بود اما سطح روی پلاسما در گروه مورد و شاهد تفاوت معنی‌داری نداشت. در هر دو گروه مورد و شاهد فراوانی بالای کمبود روی ملاحظه گردید.

Title: بررسی رابطه بین سکوت سازمانی و عملکرد سازمانی در کارکنان بیمارستان‌های آموزشی دانشگاه علوم پزشکی شهید صدوقی یزد (1393)

http://tbj.ssu.ac.ir/article-1-2269-fa.html

چکیده مقدمه: یکی از وظایف مهم مدیریت منابع انسانی در تسهیل اثربخشی یک سازمان ارزیابی عملکرد کارکنان است که تحت تاثیر عوامل مختلفی قرار می‌گیرد. یکی از این عوامل نبود فضای باز در سازمان‌ها برای بیان ایده‌های کارکنان یا همان سکوت سازمانی است. در این راستا مطالعه حاضر با هدف؛ بررسی ارتباط بین سکوت سازمانی و عملکرد سازمانی در کارکنان بیمارستان‌های آموزشی دانشگاه علوم پزشکی شهید صدوقی یزد، انجام پذیرفته است. روش بررسی: مطالعه حاضر، مطالعه‌ای توصیفی از نوع همبستگی بوده که به صورت مقطعی در سال 1393 انجام شده است. تعداد نمونه 385 نفر از کل کارکنان بیمارستان‌های آموزشی دانشگاه علوم پزشکی شهید صدوقی یزد به روش نمونه‌گیری طبقه‌ای- تصادفی انتخاب شدند. همچنین به منظور جمع‌آوری داده‌ها از پرسشنامه‌های استاندارد که روایی و پایایی آنها قبلا در مطالعات مشابه به اثبات رسیده است؛ استفاده شد. برای تحلیل داده‌ها نیز از نرم‌افزار SPSS نسخه 19 استفاده شد و با استفاده از آزمون‌های ضریب همبستگی پیرسون، آزمون T-test و Anova داده‌ها تجزیه و تحلیل شدند. یافته‌ها: در میان ابعاد سکوت سازمانی بیشترین میانگین و کمترین نمره به ترتیب مربوطه به ابعاد فرصت (98/2) و مدیریت عالی (72/2) بوده است. همچنین در میان ابعاد عملکرد سازمانی بیشترین و کمترین نمره به ترتیب مربوط به ابعاد نوآوری (27/3) و کیفیت (19/3) بوده است. بر اساس ضریب همبستگی پیرسون رابطه معنی‌داری بین دو مولفه سکوت سازمانی و عملکرد سازمانی مشاهده شد(000/0=p). نتیجه‌گیری: یافته‌های تحقیق مشخص کرد؛ رابطه معنی‌دار آماری بین دو مولفه سکوت سازمانی و عملکرد سازمانی وجود دارد، بنابراین در سازمانی که کارمندان آزادی بیان و مشارکت بیشتری در تصمیم‌گیری‌های سازمانی دارند، خود را به آن سازمان متعهد دانسته و نیز وظایف خود را به نحو شایسته‌تر، باانگیزه و با رضایت بیشتری انجام خواهند داد.

Title: بررسی عوامل مرتبط با میزان به تأخیر انداختن تولد فرزند اول (مطالعه موردی زنان همسردار 49- 15 سالۀ ساکن شهر میبد)

http://tbj.ssu.ac.ir/article-1-2270-fa.html

مقدمه: اهمیت باروری به‌عنوان مهمترین عامل تغییر در ساخت جمعیت و یکی از مؤلفه‌های اصلی رشد جمعیت موجب شده تا مطالعات مربوط به باروری، جایگاه ویژه‌ای در مطالعات جمعیت‌شناختی داشته باشد. هدف این مقاله بررسی میزان تأثیر عوامل جمعیتی، اقتصادی، اجتماعی و فرهنگی بر به تأخیر انداختن تولد نخستین فرزند در شهر میبد می‌باشد. روش بررسی: روش مطالعه در این تحقیق، پیمایشی با استفاده از ابزار پرسشنامه ساخت‌یافته است. جامعه مورد مطالعه، زنان 15 تا 49 سال ازدواج کرده و دارای حداقل یک فرزند شهر میبد و حجم نمونه شامل 379 نفر می‌باشد که به روش نمونه‌گیری تصادفی خوشه‌ای سه‌ مرحله‌ای انتخاب شده‌اند. یافته‌ها: نتایج این تحقیق نشان می‌دهد که بین متغیرهای تمایل پاسخگویان به حاملگی، نوع خانواده، سن ازدواج و مدت زمان ازدواج پاسخگویان، تحصیلات پاسخگویان و شوهران‌شان، خاستگاه جغرافیایی پاسخگویان، وضعیت شغلی زنان و نوع ازدواج آنها با متغیر وابسته میزان به‌تأخیر انداختن تولد فرزند اول رابطه وجود دارد. همچنین یافته‌های حاصل از رگرسیون چند‌متغیره حاکی از آن است که میزان استفاده از وسایل جلوگیری، سن ازدواج، خاستگاه جغرافیائی پاسخگویان، مدت ازدواج، برابری جنسیتی و نوع خانواده، به‌ترتیب بیشترین سهم را در تعیین فاصله بین ازدواج و تولد فرزند اول دارند و ‌توانسته‌اند 34 درصد از واریانس متغیر وابسته را پیش‌بینی نمایند. نتیجه‌گیری: نتایج مطالعۀ حاضر و مطالعات قبلی نشان می‌دهد که در طول سال‌های اخیر، فاصله بین ازدواج تا تولد فرزند اول افزایش یافته و از شدت باروری کاسته شده است. در مجموع از یافته‌های بدست آمده می‌توان چنین نتیجه‌گیری کرد که تأثیر شرایط جمعیتی، اجتماعی و فرهنگی در افزایش میزان به‌تأخیر انداختن تولد فرزند اول و بالطبع کاهش باروری فعلی جامعه، به‌ مراتب بیشتر از شرایط اقتصادی می‌باشد. بنابراین برای کاهش فاصله بین ازدواج تا تولد اولین فرزند در برنامه‌ریزی‌ها، باید به عوامل جمعیتی و اجتماعی– فرهنگی بیشترین توجه را نمود.

Title: بررسی رابطه بین میزان مشارکت کاری کارکنان و منابع پنجگانه قدرت مدیران در بیمارستانهای دولتی شهریزد

http://tbj.ssu.ac.ir/article-1-2272-fa.html

چکیده مقدمه: ازمهمترین اهداف هرسازمانی افزایش بهره‌وری وعملکرد بالای سازمان است و برای تحقق این اهداف نیروی انسانی نقش مهمی را ایفا می‌کند. بنابراین بایددر مورد بهبودکیفی نیروی انسانی سعی فراوان نمود چون هم به نفع سازمان وهم به نفع افرادشاغل است. برای این امر بهترین نگرش این است که چگونه توسط عوامل مختلف ازجمله استفاده ازمنابع قدرتی که دراختیارمدیر است بتوان مشارکت کاری را افزایش داد. هدف این مطالعه بررسی ارتباط بین مشارکت کاری کارکنان ومنابع قدرت مدیران دربیمارستان‌های دولتی شهریزد می‌باشد. روش بررسی: در این مطالعه توصیفی- تحلیلی، نمونه‌ای متشکل از 160 نفرازکارکنان بیمارستان‌های شهید صدوقی، شهید رهنمون، افشار و سوانح سوختگی به صورت تصادفی انتخاب گردیدند. دراین پژوهش ازدو پرسشنامه اعمال قدرت ومشارکت کاری استفاده شد. داده‌ها پس ازجمع‌آوری با استفاده ازنرم افزارspss و آزمون‌های ضریب همبستگی اسپیرمن وآزمون‌های T-test، ANOVA، من‌ویتنی وکریسکال واریس موردتجزیه وتحلیل قرارگرفت. یافته ها: 9/51 درصد از نمونه را زنان و1/48 درصد را مردان تشکیل داده‌‌اند که بیشترین درصدافراد مورد بررسی(6/50درصد) دارای مدرک لیسانس وکمترین درصدافراد(هرکدام5/2درصد)دارای دکتری عمومی و تخصصی بوده‌اند. ضریب همبستگی اسپیرمن نشان می‌دهد که قدرت قانونی بامشارکت کاری رابطه مستقیم ونسبتا قوی(05/0p< ,0<r)وقدرت اجبار و پاداش رابطه عکس(05/0p< ,0r<)و سایرقدرت‌ها رابطه معنی‌داری ندارند(05/0<p). نتیجه‌گیری:دراین تحقیق تاثیر منابع قدرت برمشارکت کاری کارکنان به ترتیب مربوط به منابع تخصص،پاداش، اجبار، مرجعیت و قانون بدست آمد. البته علی‌رغم تفاوت چشمگیر در نتایج توصیفی مشارکت کاری کارکنان بامنابع قدرت مدیران اختلاف معنی‌داری نداشت. این مقاله حاصل از پایان نامه کارشناسی مدیریت خدمات بهداشتی درمانی دانشگاه علوم پزشکی شهید صدوقی یزد می‌باشد.‌

Title: تاثیر آموزش مبتنی بر تئوری رفتار برنامه‌ریزی شده بر رفتارهای پیشگیری‌کننده از لیشمانیوز جلدی در مادران ساکن درمناطق آندمیک شهرستان نطنز

http://tbj.ssu.ac.ir/article-1-2271-fa.html

مقدمه: با توجه به مطالعات محدود در زمینه مداخلات آموزشی برای تغییر رفتارهای پیشگیری‌کننده از لیشمانیوز جلدی و همچنین نقش حساس مادران در ایجاد و ابقاء این رفتارها ، این مطالعه با هدف تعیین تاثیر آموزش مبتنی بر تئوری رفتار برنامه‌ریزی شده بر رفتارهای پیشگیری‌کننده از لیشمانیوز جلدی در مادران ساکن در مناطق آندمیک شهرستان نطنز طراحی و اجرا گردید. روش بررسی: مطالعه حاضر،مطالعه مداخله ای و از نوع تجربی می‌باشد. دو مرکز بهداشتی درمانی واقع در مناطق آندمیک بیماری لیشمانیوز جلدی به طور تصادفی به دو گروه آزمون و غیرآزمون اختصاص یافتند. سپس از لیست مادران تحت پوشش هر مرکز 80 نفر به روش نمونه‌گیری تصادفی ساده انتخاب شدند و پرسشنامه‌ای را که بر اساس تئوری رفتار برنامه‌ریزی شده طراحی شده و روایی و پایایی آن در مطالعات قبلی تایید شده بود، تکمیل نمودند. سپس 4 جلسه آموزشی برای مادران گروه آزمون و 2 جلسه آموزشی برای افراد تاثیر‌گذار بر آنها تشکیل شد ولی برای گروه غیرآزمون هیچ مداخله‌ای صورت نگرفت. 2 ماه بعد از مداخله آموزشی داده‌ها مجددا جمع‌آوری شدند وبا استفاده از نرم‌افزار Spss نسخه 18 وآزمون‌های آماری تی مستقل، تی زوجی، کای اسکوئر و من ویتنی مورد تجزیه و تحلیل قرار گرفتند. یافته‌ها: قبل از مداخله تفاوت معنی‌داری بین میانگین نمرات سازه‌های مختلف این تئوری در دوگروه مشاهده نشد)005/0<P ( ولی بعد از مداخله در میانگین نمرات آگاهی، نگرش، هنجارهای انتزاعی،کنترل رفتاری درک شده، قصد و عملکرد گروه آزمون افزایش معنی‌دار مشاهده شد (005/0>P) و در گروه غیر آزمون فقط در میانگین نمرات آگاهی و نگرش افزایش معنی‌دار مشاهده شد (005/0>P). نتیجه‌گیری: آموزش بر اساس تئوری رفتار برنامه‌ریزی شده می‌تواند رفتارهای پیشگیری‌کننده از لیشمانیوز جلدی را در مادران ارتقا‌ بخشد.

Title: رابطه ابعاد سرمایه اجتماعی و سلامت درک شده در جامعه شهری یزد، 1392

http://tbj.ssu.ac.ir/article-1-2273-fa.html

مقدمه: سلامت، اساسی‌ترین جزء رفاه جامعه به شمار می‌رود و بیش از مداخلات پزشکی به عوامل اجتماعی وابسته است. مفهوم سرمایه اجتماعی به دلیل ماهیت و محتوایی که دارد تقریباً با تمامی موضوعات و مسائل مطرح در حوزه انسانی، اجتماعی و بهداشتی ارتباط پیدا می‌کند. سرمایه اجتماعی و مسائل اجتماعی ارتباط تنگاتنگی با سلامت دارند. لذا، هدف پژوهش حاضر، بررسی رابطه ابعاد سرمایه اجتماعی و سلامت درک شده در جامعه شهری یزد می‌باشد. روش بررسی: این پژوهش از نوع تحلیلی و به‌صورت مقطعی می‌باشد. جامعه موردپژوهش شامل کلیه شهروندان یزد می‌باشد. تعداد حجم نمونه برابر با 380 نفر است که با روش نمونه‌گیری خوشه‌ای تصادفی انتخاب گردیده‌اند. ابزار گردآوری داده‌ها، پرسشنامه بود که شامل دو پرسشنامه سرمایه اجتماعی و سلامت درک شده است. یافته­ها: در بین ابعاد سرمایه اجتماعی؛ احساس اعتماد و امنیت، کیفیت ارتباطات فامیل و دوستان، ارزش زندگی، تساهل در زمینه تنوع فرهنگی و کیفیت ارتباطات کاری با سلامت درک شده رابطه مستقیم و معناداری وجود دارد اما بین مشارکت در فعالیت‌های اجتماع محلی، عامل بودن در بستر اجتماعی و کیفیت ارتباطات بین همسایه‌ها با سلامت درک شده رابطه معناداری وجود ندارد. نتیجه­گیری: سرمایه اجتماعی و ابعاد آن با سلامت درک شده رابطه مستقیم و معناداری دارد؛ بنابراین، با افزایش سرمایه اجتماعی، میزان سلامت درک شده نیز بیشتر می‌شود.

Title: استرس و پیشگویی‌کننده‌های سلامت روان در نوجوانان ایرانی

http://tbj.ssu.ac.ir/article-1-2275-fa.html

چکیده مقدمه: امروزه استرس یکی از علل عمده بسیاری از بیماری‌ها محسوب می‌شود و دوران نوجوانی به دلیل مصادف شدن با بلوغ، دوران استرس و فشارهای روانی- اجتماعی می‌باشد. شمار زیادی از نوجوانان از استرس رنج می‌برند نظر به اینکه یک‌سوم جمعیت ایران را نوجوانان تشکیل می‌دهند این مطالعه باهدف بررسی پیش‌بینی کننده‌های استرس در دانش آموزان مقطع دبیرستان انجام گردید. روش بررسی: این مطالعه از نوع مطالعات توصیفی - مقطعی بود و تعداد 403 نفر از دانش آموزان دختر و پسر مقاطع دبیرستان از 4 مدرسه به‌طور تصادفی انتخاب و در مطالعه بر اساس توزیع سهمیه‌ای وارد گردیدند. داده‌های مطالعه بر اساس پرسشنامه‌های دموگرافیک، سلامت عمومی گلدبرگ، خودکارآمدی شرر، رضایت از زندگی دینر، استرس کوهن، امیدواری اشنایدر و شادکامی اکسفورد به‌صورت خود گزارش دهی جمع‌آوری گردیدند. بعد از جمع‌آوری اطلاعات و واردکردن داده‌ها در نرم‌افزار آماری 16- SPSS با استفاده از آزمون‌های لوجستیک رگرسیون و همبستگی مورد تجزیه‌وتحلیل قرار گرفتند. یافته‌ها: نتایج مطالعه نشان داد که 55% از دختران و 3/46% از پسران دچار استرس بالابودند و 62% از دانش آموزان از مشکلات روان رنج می‌بردند. همچنین با افزایش نمره استرس احتمال رخداد مشکل سلامت روان در دانش آموزان به میزان 27% افزایش می‌یابد. نتیجه‌گیری: یافته‌های پژوهش حاضر نشان داد که اکثر دانش آموزان از مشکل سلامت روان رنج می‌برند و استرس یکی از مهم‌ترین فاکتورهای پیشگویی‌کننده سلامت روان می‌باشد.

Title: بررسی عوامل موثر بر عفونت ادراری در بارداری

http://tbj.ssu.ac.ir/article-1-2274-fa.html

مقدمه: زنان بسیار مستعد ابتلا به عفونت ادراری می‌باشند و حاملگی شانس ابتلا به عفونت ادراری را بالا می‌برد. درمجموع اطلاعات اندکی از عوامل خطر عفونت ادراری در بارداری در دست است. عفونت ادراری در بارداری عامل خطر مهمی برای پیامدهای وخیم بارداری محسوب می‌گردد. هدف از این مطالعه یافتن عوامل خطر مرتبط با عفونت ادراری در زنان باردار است. روش بررسی: این مطالعه از نوع مشاهده‌ای تحلیلی و به‌صورت گذشته‌نگر در زمستان 1393 در شهرکرد انجام شد؛ که در آن تعداد 310 زن باردار مراجعه‌کننده به یازده مرکز بهداشتی درمانی شهرکرد شرکت داشتند. از این تعداد155 نفر مورد (بیمار) و 155 نفر شاهد (سالم) بودند که ازنظر سن باهم همسان‌سازی شده بودند اطلاعات موردنیاز از طریق پرونده بهداشتی مادران باردار و تکمیل چک‌لیست محقق ساخته‌ای که اعتبار آن توسط متخصصین تأییدشده بود جمع‌آوری شد. چک‌لیست شامل تعدادی از عوامل خطر احتمالی بیماری و همچنین خصوصیات دموگرافیک شرکت‌کنندگان در مطالعه بود. تجزیه‌وتحلیل آماری توسط نرم‌افزار spss نسخه 16 و با استفاده از آزمون‌های آماری مجذور کای و تی مستقل و رگرسیون لوجستیک انجام شد. یافته‌ها: از میان متغیرهای موردبررسی استفراغ بارداری(0.00p=), سابقه عفونت ادراری در بارداری قبلی (4.408-1.508Cl=،0.01p=،2.587OR=))وسابقه سقط خود به خودی(3.847-1.165Cl=،0.014p=،2.117OR=)به ترتیب مهم‌ترین عوامل خطر ابتلا به عفونت ادراری در زنان باردار تعیین گردید. نتیجه‌گیری: پیشگیری و درمان استفراغ بارداری پیشگیری از ابتلا به عفونت ادراری در طول بارداری پیشین و پیشگیری از سقط‌ها می‌تواند نقش مهمی در پیشگیری از ابتلا به عفونت ادراری و عوارض ناشی از آن داشته باشد. همچنین این مطالعه نشان داد که تعدادی از عوامل می‌تواند بر روی عفونت ادراری در بارداری تأثیر داشته باشد که تاکنون موردتوجه کافی نبوده‌اند و لازم است که در برنامه‌های بهداشتی و پیشگیری موردتوجه بیشتر قرار گیرند.

Title: بررسی تاثیر فرایند کمپوست سازی ضایعات فراوری پسته با تیمارهای مختلف بر غلظت فلزات سنگین

http://tbj.ssu.ac.ir/article-1-2276-fa.html

چکیده مقدمه: یکی از روش­های مدیریت ضایعات فراوری پسته کمپوست کردن می­باشد. در تولید کود کمپوست مناسب، عناصر غذایی و فلزات سنگین تعیین‌کننده می­باشد. هدف این مطالعه بررسی تأثیر فرایند کمپوست سازی ضایعات فراوری پسته با تیمارهای مختلف بر غلظت فلزات سنگین است. روش بررسی: در این تحقیق ضمن انجام فرایند کمپوست­سازی ضایعات فراوری پسته با دو تیمار کود گاوی و لجن آبگیری شده فاضلاب شهری، شاخص­های فلزات سنگین و مواد مغذی طی فرایند 60 روزه موردبررسی قرار گرفت نتایج به‌دست‌آمده با استاندارد ملی ایران و WHO مقایسه گردید. ترسیم نمودارها توسط نرم‌افزار excel نسخه 2007 و تجزیه‌وتحلیل با استفاده از نرم­افزار آماری Spss (نسخه 20) در سطح معناداری 05/0 انجام شد. یافته­ها: طی فرایند 60 روزه تولید کمپوست pH ابتدا روند کاهشی داشته و سپس افزایش پیدا کرد. میزان Cu، Zn، Fe، Mn و نسبت C/N روند کاهشی و میزان EC، Na، K روند افزایشی داشتند. درنهایت میزان آهن، روی، مس و منگنز کمتر از استاندارد، سدیم در حد استاندارد و پتاسیم بیشتر از استانداردهای تعیین‌شده در کمپوست تولیدی از ضایعات فراوری پسته با هر دو تیمار بود. نتیجه­گیری: نتایج این بررسی نشان داد غلظت فلزات سنگین و عناصر غذایی در کمپوست تولیدی با هر دو تیمار در محدوده قابل‌قبول قرار داشت. درنهایت کیفیت کمپوست تولیدی با تیمار کود گاوی به علت تجزیه بهتر درنتیجه پایداری بیشتر آن بهتر از کمپوست تولیدی با تیمار لجن آبگیری شده فاضلاب شهری بود.         این مقاله برگرفته از پایان نامه  دانشجویی دانشگاه علوم پزشکی شهید صدوقی یزد می باشد.

Title: بررسی موانع موثر بر رفتارهای خود مراقبتی بیماران دیابتی نوع 2 استان یزد، در سال 1393

http://tbj.ssu.ac.ir/article-1-2278-fa.html

مقدمه: مبتلایان به بیماری دیابت ممکن است در انجام صحیح رفتارهای خودمراقبتی و پایبندی به آن ضعف داشته باشند، بنابراین شناسایی موانع خودمراقبتی اولین گام برای بهبود و ارتقای رفتارهای مراقبت از خود می باشد. این پژوهش با هدف تعیین موانع موثر بر رفتارهای خودمراقبتی  بیماران دیابتی نوع 2 در شهر یزد انجام گرفته است. روش بررسی: مطالعه حاضر مطالعه ای توصیفی - همبستگی از نوع مقطعی است. 152 بیمار مبتلا به دیابت نوع 2 مراجعه کننده به مرکز تحقیقات دیابت استان یزد، به روش سیستماتیک، مورد بررسی قرار گرفتند. ابزار جمع آوری داده ها، پرسشنامه پژوهشگر ساخته می باشد که روایی و پایایی آن بررسی شد و میزان آلفای کرونباخ  برای این پرسشنامه 8/0 بدست آمد. داده ها با استفاده از نرم افزار SPSS نسخه 16 و آزمون های آماری t مستقل، آنالیز واریانس یک طرفه و ضریب همبستگی پیرسون مورد تجزیه و تحلیل قرار گرفت. یافته­ها: امتیاز موانع انجام رفتارهای خودمراقبتی بیماران دیابتی از 100 نمره 03/52  بود که بر اساس سطح مطلوبیت تعیین شده و در نظر گرفتن دامنه امتیاز موانع، در سطح متوسط ارزیابی شد. نتایج حاکی از آن بود که موانع اقتصادی بیشترین سهم را در بین موانع خودمراقبتی به خود اختصاص داده اند ( 73 امتیاز) و موانع  دانش و نگرش(63 امتیاز)، در ردیف دوم قرار گرفت. نوع موانع موثر در خودمراقبتی بر حسب سن، جنس، تحصیلات، وضعیت اشتغال، میزان درآمد، مدت ابتلا به دیابت، ابتلا به عوارض دیابت و  مراجعه طبق توصیه پزشک  به طور معناداری متفاوت بود )05/0( p<. نتیجه­گیری: شناسایی موانع خودمراقبتی، خصوصا بعد اقتصادی،از گام های مهم در کمک به بیماران دیابتی می باشد به طوری که اقداماتی چون قرار دادن آزمایشاتی نظیر پایش قندخون بیماران دیابتی، تحت پوشش بیمه خدمات درمانی و تامین ابزارها و تجهیزات خودپایشی و خودمراقبتی سبب بهبود خودمراقبتی خواهد شد.

Title: ارزیابی کارایی صافی های دو لایه ای در بهبود کیفیت آب در تصفیه خانه ی شهر آبادان

http://tbj.ssu.ac.ir/article-1-2280-fa.html

چکیده مقدمه: امروزه، بیشترین نوع فرایندی که در تصفیه‌ی آب آشامیدنی استفاده می‌شود فرایند تصفیه معمولی و متداول است که نمی‌تواند به‌طور مؤثری مواد آلی را حذف کند. دفیلتراسیون، یکی از مهم‌ترین فرایندهای تصفیه استفاده‌شده در تصفیه‌خانه آب می‌باشد. یکی از راهکارها، بهسازی فیلتر ماسه‌ای سریع به فیلتر دو یا چند لایه‌ای می‌باشد. روش بررسی: این مطالعه، نتایج حاصل از یک تحقیق آزمایشگاهی در مقیاس پایلوت بود که باهدف بررسی عملکرد استفاده از GAC و آنتراسیت در صافی دولایه‌ای در تصفیه‌خانه‌ی آب آبادان در حذف پارامترهای کدورت، TOC، آهن، منگنز، نیترات و آمونیوم در واحد فیلتراسیون تصفیه‌خانه‌ی آب آبادان موردبررسی قرار گرفت. کلیه‌ی آزمایش‌ها به روش استاندارد انجام شد. نتایج با استفاده از آزمون‌های آماری، موردبررسی قرار گرفت. یافته‌ها: نمونه‌ها در فصل تابستان از ورودی و خروجی پایلوت‌ها و واحد فیلتراسیون تصفیه‌خانه جمع‌آوری شدند و پارامترهای مذکور در هر نمونه طی سه بارگذاری کم، متوسط و زیاد موردسنجش قرار گرفتند. پایلوت شماره دو بیشترین راندمان را در حذف پارامترهای موردنظر داشته است که میزان حذف پارامترهای کدورت، TOC، آهن، منگنز، نیترات و آمونیوم به ترتیب 79/89%، 67/91%، 43/50%، 67/17%، 42/35% و 33/42% بوده است. همچنین بهترین عملکرد فیلتر GAC-sand در EBCT=20min طی بار سطحی 35 l/d می‌باشد. نتیجه‌گیری: به‌طورکلی، هر دو پایلوت در بارگذاری کم راندمان بالاتری دارند به‌خصوص پایلوت شماره دو نشان داد که به علت EBCT بالاتر، کارایی بیشتری در حذف پارامترهای یادشده دارد.

Title: بررسی فراوانی مصرف مکمل های غذایی در بیماران مبتلا به آرتریت روماتوئید مراجعه کننده به بیمارستان های دولتی شهر یزد

http://tbj.ssu.ac.ir/article-1-2281-fa.html

مقدمه: بیماری آرتریت روماتوئید (Rheumatoid Arthritis)(RA) یکی از بیماریهای خودایمنی است که بیگانه انگاشتن ترکیبات مختلف مفاصل از جمله خصوصیات این بیماری است. بر اساس یافته های علمی، گستره وسیعی از روشهای درمانی مکمل و جایگزین (شامل مکمل های گیاهی و غذایی) برای روماتیسم مفصلی مفیدند. روش بررسی: مطالعه ی مقطعی برروی120 نفرازافراد مبتلا به آرتریت روماتوئید مراجعه کننده به مراکز درمانی دولتی در شهر یزد در طی سال1393 انجام گردید. با استفاده از پرسشنامه طراحی شده، شاخص های دموگرافیک و نوع میزان مصرف مکمل های مورد مطالعه در بیماران مبتلاء به آرتریت روماتوئید مورد ارزیابی قرار گرفت. یافته ها: از بین 120 بیمار موردمطالعه، 118 نفرحداقل یکی از مکمل های مورد نظر را استفاده می کردند. افراد درسنین بین 76-19 سال قرار داشتند که 6/71 درصد آنها بالای 40 سال سن داشتند. 85% افراد مورد مطالعه زن، 92% متاهل، 55% درآمد بین 1 تا 2 میلیون تومان و 7/51% سطح سوادشان دیپلم و بالاتر بود. بیشترین میزان مصرف مکمل مربوط به کلسیم- ویتامین د (7/90%) بود. اسید فولیک (5/80%)، ویتامین D(7/73%) و امگا 3(9/55%) در ردیفهای بعدی مصرف قرار داشتند. لازم به ذکر است که اکثر بیماران (5/96%) زیر نظرپزشک متخصص روماتولوژِی مکمل ها را مصرف می کردند. نتیجه گیری: باتوجه به دادههای به دست آمده مبنی بر استفاده از مکمل های مختلف در بیماران آرتریت روماتوئید، بیشترین مکمل های مورد استفاده شامل کلسیم- ویتامین د، اسیدفولیک ویتامین د و امگا 3 بود. اکثر بیماران از تأثیرمثبت این مکمل ها راضی بودند.

Title: بررسی شیوع افسردگی، اضطراب و استرس در جمعیت شهر یزد در سال 1392

http://tbj.ssu.ac.ir/article-1-2284-fa.html

چکیده مقدمه: افسردگی و اضطراب با سرعت شیوع 20-10 درصد در سال در جمعیت عمومی از شایع‌ترین اختلالات روان‌پزشکی می‌باشد. وجود آرامش و اجتناب از افسردگی، اضطراب و استرس موجب بالا رفتن سطح رفاه و بهزیستی جامعه می‌شود. مطالعه حاضر با هدف بررسی شیوع افسردگی، اضطراب و استرس در شهر یزد انجام شد. روش بررسی: مطالعه حاضر از نوع توصیفی- تحلیلی است که بر روی 400 نفر از جمعیت شهر یزد در سال 1392 انجام شد. نمونه‌گیری به روش خوشه‌ای انجام شد و 16 خوشه 25  خانواری بررسی شدند. داده‌ها با استفاده از پرسشنامه استاندارد DASS-21 جمع‌آوری گردید. جهت تجزیه و تحلیل داده‌ها از آزمون‌های ناپارامتریک Mann-whitney، Kruskal-wallis و رگرسیون لجستیک با نرم افزار SPSS نسخه 16 استفاده شد. یافته‌ها: میزان شیوع افسردگی در مطالعه حاضر 3/54 درصد، میزان شیوع اضطراب 7/57 درصد و میزان شیوع استرس 51 درصد بود. نتایج بدست آمده از آنالیز رگرسیون لجستیک نشان داد سابقه داشتن بیماری مزمن و میزان درآمد پایین متغیرهای پیش‌بینی‌کننده افسردگی بودند. همچنین جنسیت، میزان تحصیلات پایین، سابقه داشتن بیماری مزمن و میزان درآمد پایین متغیرهای پیش‌بینی‌کننده اضطراب و نوع شغل، استفاده از سیگار و سابقه داشتن بیماری مزمن متغیرهای پیش‌بینی‌کننده استرس بودند. نتیجه گیری: نتایج پژوهش حاکی از بالا بودن افسردگی، اضطراب و استرس در جمعیت مورد مطالعه بود و در بین آنها اضطراب دارای شیوع بالاتری بود. بنابراین رسیدگی به این مشکل و برنامه‌ریزی برای بهبود شرایط حائز اهمیت می‌باشد. افزایش آگاهی مردم، افزایش مراکز مشاوره مخصوصاً مشاوره تلفنی و بهبود اماکن تفریحی و ورزشی در این راستا حائز اهمیت است.

Title: اثر فرایندهای حرارت دهی و انواع طبخ خانگی برنج بر غلظت آفلاتوکسین B1

http://tbj.ssu.ac.ir/article-1-2282-fa.html

مقدمه: آلودگی برخی محصولات کشاورزی به مایکوتوکسین ها سلامت انسان و حیوان را با نگرانی جدی روبه‌رو کرده است. آفلاتوکسین  B1 سمی‌ترین نوع آفلاتوکسین است که در بین آفلاتوکسین ها دارای بیشترین میزان شیوع در غذاها ازجمله برنج می‌باشد. هدف از انجام این تحقیق، مطالعه اثر فرایندهای حرارت دهی و انواع طبخ خانگی برنج بر میانگین غلظت آفلاتوکسین  B1می‌باشد. روش بررسی: در این مطالعه فرایندهای حرارت دهی 20 و 120 دقیقه و انواع طبخ به سه صورت آبکشی، کته و برنج بوداده بر روی برنج به‌صورت طبیعی آلوده موردبررسی انجام گرفت و مقدار آفلاتوکسین  B1با روش کروماتوگرافی مایع با کارایی بالا (HPLC) اندازه‌گیری شد. یافته‌ها: در مورد 20 دقیقه و 120 دقیقه حرارت دهی برنج میانگین غلظت آفلاتوکسین  B1 به میزان به ترتیب 6/16% و 7/81% کاهش نشان داد. اگرچه این میزان کاهش ازنظر آماری در 20 دقیقه حرارت دهی معنی‌دار نبود اما در 120 دقیقه حرارت دهی کاهش معناداری (05/0p <) مشاهده شد. میزان کاهش میانگین غلظت آفلاتوکسین  B1 در پخت کته، آبکشی و برنج بوداده به ترتیب 2/60%، 5/85% و 6/87% نشان داده شد که این میزان کاهش در پخت کته ازنظر آماری معنی‌دار نشد ولی در پخت به‌صورت آبکشی و برنج بوداده این میزان کاهش ازنظر آماری با (05/0p <) معنی‌دار بود. نتیجه‌گیری: نتایج این مطالعه نشان می‌دهد که انجام فرایندهای حرارت دهی و انواع طبخ برنج می‌تواند خطرات سم آفلاتوکسین  B1 که انسان به‌وسیله مصرف برنج با آن مواجه است را کاهش دهد.

Title: بررسی سطح سواد سلامت در مراجعین به مرکز تحقیقات دیابت یزد

http://tbj.ssu.ac.ir/article-1-2283-fa.html

مقدمه:توانایی به کار گیری مهارت هایی نظیر خواندن، شنیدن، تجزیه و تحلیل و تصمیم گیری مناسب در موقعیت های سلامتی مرهون میزان ظرفیت هر فرد برای کسب، تفسیر و درک اطلاعات اولیه و خدمات سلامتی است که در قالب سواد سلامت فرد تعریف می شود. هدف از مطالعه حاضر تعیین سطح سواد سلامت در مراجعین به مرکز تحقیقات دیابت یزد بوده است. روش بررسی: این مطالعه به روش مقطعی و تحلیلی  بر روی 432 بیمار مراجعه کننده به مرکز تحقیقات دیابت شهر یزد در سال 1393 انجام گردید. داده ها با استفاده از پرسشنامه استاندارد سواد سلامتی عملکردی بزرگسالان(Test Of Functional Health Literacy in Adults) TOFHLA جمع آوری و در نرم افزار SPSS نسخه 17 با استفاده از آزمون T-Test، Chi-Square، A-Nova و رگرسیون خطی مورد تجزیه و تحلیل قرار گرفتند. یافته ها: میانگین سنی افراد مورد مطالعه 32/6 ± 02/55 سال و میانگین سابقه ابتلا به دیابت 13/7 ± 24/10 سال می باشد. افزایش نمره سواد سلامت با افزایش سن و کاهش سال های ابتلا به دیابت همراه است. میانگین نمره سواد سلامت به صورت معنی داری در آقایان بیشتر از خانم ها و در افراد بازنشسته، افراد ساکن شهر، افراد با میزان تحصیلات راهنمایی تا دیپلم و دانشگاهی و دارای وضعیت اقتصادی خوب و متوسط بیشتر از سایرین می باشد. (001/0P <). نتیجه گیری:نتایج تحقیق حاضر نشان داد که سواد سلامت در 3/59% بیماران دیابتی مراجعه کننده به مرکز تحقیقات دیابت، ناکافی و در 5/18% مرزی بود و فقط 2/22% افراد سواد سلامت کافی داشتند که لزوم ایجاد تسهیلات جهت آموزش سواد سلامت در این بیماران ضروری به نظر می رسد.

Title: بررسی الگوی مقاومت آنتی‌بیوتیکی سویه های کلبسیلا پنومونیه جداشده از نمونه‌های ادراری در بیمارستان‌های آموزشی دانشگاه علوم پزشکی شهید صدوقی یزد

http://tbj.ssu.ac.ir/article-1-2286-fa.html

چکیده مقدمه: کلبسیلا پنومونیه یک پاتوژن فرصت‌طلب و یکی از عوامل مهم ایجادکننده عفونت‌های ادراری، داخل شکمی و پنومونی در بیماران دارای ضعف سیستم ایمنی با بیماری‌های زمینه‌ای است. استفاده بی‌رویه و نامنظم از آنتی‌بیوتیک‌ها باعث ایجاد مقاومت آنتی‌بیوتیکی می‌گردد. سازمان بهداشت جهانی مقاومت باکتری‌ها به آنتی‌بیوتیک‌ها را جزء مهم‌ترین خطرات تهدیدکننده بهداشت جهانی معرفی کرده است. در این مطالعه الگوی مقاومت آنتی‌بیوتیکی سویه های کلبسیلا پنومونیه جداشده از نمونه‌های ادراری بررسی شد. روش بررسی: در این مطالعه توصیفی- مقطعی، تعداد 130 ایزوله کلبسیلا پنومونیه از عفونت‌های ادراری بیماران بستری در بیمارستان‌های دانشگاهی یزد جمع‌آوری و با آزمایش‌های بیوشیمیایی شناسایی شدند. سنجش حساسیت آنتی‌بیوتیکی به روش استاندارد انتشار از دیسک طبق پروتکل CLSI انجام و نتایج با نرم‌افزارSPSS نسخه 16 آنالیز شد. یافته‌ها: از 130 سویه کلبسیلا پنومونیه، 72 سویه (4/55 %) از بیماران مونث جدا شد. بیشترین مقاومت آنتی‌بیوتیکی به ترتیب نسبت به آنتی‌بیوتیک‌های آموکسی سیلین (100 %) و سفوتاکسیم (5/41 %) و کمترین میزان مقاومت نسبت به مروپنم (8/3 %) و ارتاپنم (8/3 %) مشاهده شد. نتیجه‌گیری: نتایج مطالعه حاضر و مطالعات مشابه نشان می‌دهند که متأسفانه مقاومت به آنتی‌بیوتیک‌های مختلف رو به افزایش می‌باشد، لذا توصیه می‌شود قبل از درمان بایستی سنجش حساسیت ضد میکروبی انجام شود و سالیانه الگوی مقاومت آنتی‌بیوتیکی بررسی و نتایج به پزشکان و کمیته‌های کنترل عفونت گزارش گردد. این مقاله برگرفته از پایان‌نامه کارشناسی ارشد میکروب‌شناسی پزشکی مصوب دانشگاه علوم پزشکی شهید صدوقی یزد می‌باشد.

Title: بررسی میزان تاثیرکارگاه آموزشی مهارت‌های ارتباطی بر مؤلفه‌های فرسودگی شغلی، در پرستاران شاغل در بیمارستان‌های وابسته به دانشگاه علوم پزشکی تهران

http://tbj.ssu.ac.ir/article-1-2285-fa.html

مقدمه: پژوهش حاضر باهدف بررسی میزان تاثیر کارگاه آموزشی مهارت‌های ارتباط موثر بر مؤلفه‌های فرسودگی شغلی پرستاران شاغل در بیمارستان‌های وابسته به دانشگاه علوم پزشکی تهران انجام‌شده است. روش بررسی: این پژوهش یک مطالعه مداخله‌ای روی ١۰۷ نفر از پرستاران شاغل در بیمارستان‌های وابسته به دانشگاه علوم پزشکی تهران انجام شد. نمونه‌گیری به صورت خوشه‌ای ساده از بین بیمارستان‌های دانشگاه علوم پزشکی تهران صورت گرفت و شرکت‌کنندگان به روش تصادفی به دو گروه مداخله و غیر مداخله تقسیم شدند. شرکت‌کنندگان پرسشنامه‌ی دو قسمتی متغیرهای زمینه‌ای و فرسودگی شغلی مسلچ (MBI) را تکمیل کردند. سپس گروه مداخله در کارگاه آموزشی مهارت‌های ارتباط موثر شرکت داده شدند. سه ماه پس از آموزش، مجددا شرکت‌کنندگان پرسشنامه‌ی فرسودگی شغلی را تکمیل کردند. داده های به دست آمده با نرم افزار ١٨ Spss مورد تجزیه‌وتحلیل قرار گرفت. یافته‌ها: نتایج حاصل از آزمون‌های آماری t زوجی در گروه مداخله، قبل و بعد از آموزش نشان داد که در میانگین نمره مؤلفه خستگی عاطفی در مقیاس فراوانی (008/0>p ) و شدت (001/0> p) و همچنین میانگین مؤلفه مسخ شخصیت در مقیاس شدت (03/0> p) تفاوت معنی‌دار آماری وجود دارد. همچنین میانگین نمره هر سه مؤلفه فرسودگی شغلی در هر دو زیر مقیاس فراوانی و شدت، در هر دو گروه مداخله و غیر مداخله بعد از آموزش اختلاف معنی‌دار آماری داشته است (05/0> p) که نشان دهنده تاثیر گذاری دوره آموزشی بر فرسودگی شغلی پرستاران شاغل در بیمارستان می‌باشد. نتیجه‌گیری: با آگاهی از تأثیرگذار بودن دوره آموزشی مهارت‌های ارتباط موثر بر مؤلفه‌های فرسودگی شغلی، می‌توان برای برنامه‌های پیشگیری و ارتقاء سلامت در پرستاران استفاده کرد.

Title: حذف 4-کلرفنل از فاضلاب سنتتیک با استفاده از گرانول های نانو ذرات اکسید گرافن

http://tbj.ssu.ac.ir/article-1-2287-fa.html

چکیده  مقدمه : 4-کلروفنل یکی از آلاینده­های رایج در فاضلاب تولیدی از صنایع شیمیائی و پتروشیمی می­باشد. این ماده بدلیل حضور کلر و حلقه بنزنی در آن مقاوم در برابر تجزیه بیولوژیکی می باشد. در این  پژوهش، گرانول های نانو ذرات اکسید گرافن بعنوان یک جاذب نانو ساختار با خواصی بهتر نسبت به سایر جاذب­ها جهت حذف4-کلروفنل از محیط آبی مورد بررسی قرارگرفت. روش بررسی:این پژوهش در سال 93-92 انجام گردید که در آن با استفاده از سیلیکات سدیم نانو ذرات اکسید گرافن گرانول سازی  و جهت شناسایی مورفولوژی و ساختار جاذب از روش‌های پراش سنجی پرتو ایکس و تصویر میکروسکوپ پراش الکترونیکی استفاده شد. متغیر­های موثر بر فرایند جذب از جمله غلظت اولیه   4-کلرو فنل(5 ،10 ،15 ، 20 و25 میلی گرم بر لیتر)، مقدار جاذب(20 ، 40 ، 60 و 80 درصد وزنی) ،زمان تماس( 3، 5، 10، 20و 30دقیقه)  وpH 4 تا 9 به روش تغییر یک عامل در زمان بررسی گردید. در انتها داده­ها با ایزوترم های لانگمویر و فروندلیچ مطابقت داده شد. یافته ها: فرایند جذب در زمان 20 دقیقه به تعادل رسید. مقدار2گرم برلیتر گرانول اکسید گرافن در زمان 3 دقیقه و7=pH توانست 5 میلی گرم بر­لیتر 4-کلروفنل را تا58%حذف نماید. ایزوترم فروندلیچ فرایند جذب را بخوبی توصیف نمود. مقدارR2 ، 92/0 بدست آمد. نتیجه گیری: فرایند جذب بوسیله جاذب گرانول های نانو ذرات اکسید گرافن از فرایندهای ارزان قیمت با راندمان بالا می­باشد و می­توان از آن درکاهش و از بین بردن آلاینده­های محیطی و ‌بویژه محیط های آبی استفاده نمود.

Title: بررسی فناوری‌های نوین در حذف آفت‌کش آترازین از محیط زیست با تأکید بر تجزیۀ‌زیستی: یک مطالعه مروری

http://tbj.ssu.ac.ir/article-1-2288-fa.html

مقدمه: با کمبود زمین‌های زراعی و از بین رفتن محصولات بوسیلۀ آفات استفاده از آفت‌کش‌هایی مانند آترازین افزایش یافته است. این علف‌کش به دلیل فشار بخار کم، نیمه عمر بالا و تحرک زیاد منجر به آلودگی اکوسیستم‌های گوناگون شده است. آترازین از نظر سمیت در کلاس III بوسیلۀ سازمان حفاظت محیط زیست آمریکا قرار گرفته است، اما به دلیل توانایی بالقوۀ آن در آلودگی آب‌های زیرزمینی اهمیت بسیاری دارد. روش بررسی: روش‌های فیزیکی و شیمیایی بسیاری برای حذف این علف‌کش از محیط‌های آبی و خاکی پیشنهاد گردیده، اما این روش‌ها دارای هزینه‌های بسیار و همراه با تولید محصولات جانبی سمی دیگری هستند. با توجه به گسترش علم تعامل انسان و طبیعت، امروزه تصفیۀ بیولوژیکی از اهمیت و توجه خاصی برخوردار است. تصفیۀ بیولوژیکی فرآیندی است که در آن از میکروارگانیزم‌ها برای تبدیل و تجزیۀ مواد آلاینده موجود در محیط زیست استفاده می‌شود. نتیجه‌گیری: تجزیۀ زیستی از نقطه نظر اقتصادی و زیست محیطی بهترین راهکار برای حذف آلاینده‌های دیرپا از محیط زیست می‌باشد. امروزه استفاده از میکروارگانیسم‌های بومی یا اصلاح شده بوسیلۀ مهندسی ژنتیک برای تصفیۀ محیط‌های آلوده به سموم آفت‌کش بطور فزاینده‌ای مورد استفاده قرار می‌گیرد. در پایان به منظور کاربرد بیشتر این فناوری سبز تعیین خصوصیات خاک و همچنین مطالعات سم‌شناسی محیطی (اکوتوکسیکولوژی) با هدف شناسایی توانایی میکروارگانیسم‌های بومی آب و خاک یک امر ضروری می‌باشد.
